# Identification of the enhancer RNAs related to tumorgenesis of pituitary neuroendocrine tumors

**DOI:** 10.3389/fendo.2023.1149997

**Published:** 2023-07-18

**Authors:** Liangbo Wang, Chenlu Wei, Yu Wang, Ning Huang, Tao Zhang, Yuting Dai, Li Xue, Shaojian Lin, Zhe Bao Wu

**Affiliations:** ^1^Department of Neurosurgery, First Affiliated Hospital of Wenzhou Medical University, Wenzhou, China; ^2^Department of Neurosurgery, Ruijin Hospital, Shanghai Jiao Tong University School of Medicine, Shanghai, China; ^3^Center for Reproductive Medicine, the First Affiliated Hospital of Zhengzhou University, Zhengzhou, China; ^4^Department of Neurosurgery, Ren Ji Hospital, School of Medicine, Shanghai Jiao Tong University, Shanghai, China; ^5^State Key Laboratory of Medical Genomics, Shanghai Institute of Hematology, National Research Center for Translational Medicine at Shanghai, Ruijin Hospital, Shanghai Jiao Tong University School of Medicine, Shanghai, China

**Keywords:** pituitary neuroendocrine tumors (PitNETs), enhancer RNAs, prediction model, regulatory network, ciclopirox

## Abstract

**Background:**

Pituitary neuroendocrine tumors (PitNETs), which originate from the pituitary gland, account for 10%–15% of all intracranial neoplasms. Recent studies have indicated that enhancer RNAs (eRNAs) exert regulatory effects on tumor growth. However, the mechanisms underlying the eRNA-mediated tumorigenesis of PitNETs have not been elucidated.

**Methods:**

Normal pituitary and PitNETs tissues were used to identify the differentially expressed eRNAs (DEEs). Immune gene sets and hallmarks of cancer gene sets were quantified based on single sample gene set enrichment analysis (ssGSEA) algorithm using GSVA. The perspective of immune cells among all samples was calculated by the CIBERSORT algorithm. Moreover, the regulatory network composed of key DEEs, target genes of eRNAs, hallmarks of cancer gene sets, differentially expressed TF, immune cells and immune gene sets were constructed by Pearson correlation analysis. Small molecular anti-PitNETs drugs were explored by CMap analysis and the accuracy of the study was verified by *in vitro* and *in vivo* experiments, ATAC-seq and ChIP-seq.

**Results:**

In this study, data of 134 PitNETs and 107 non-tumorous pituitary samples were retrieved from a public database to identify differentially expressed genes. In total, 1128 differentially expressed eRNAs (DEEs) (494 upregulated eRNAs and 634 downregulated eRNAs) were identified. Next, the correlation of DEEs with cancer-related and immune-related gene signatures was examined to establish a co-expression regulatory network comprising 18 DEEs, 50 potential target genes of DEEs, 5 cancer hallmark gene sets, 2 differentially expressed transcription factors, 4 immune cell types, and 4 immune gene sets. Based on this network, the following four therapeutics for PitNETs were identified using Connectivity Map analysis: ciclopirox, bepridil, clomipramine, and alexidine. The growth-inhibitory effects of these therapeutics were validated using *in vitro* experiments. Ciclopirox exerted potential growth-inhibitory effects on PitNETs. Among the DEEs, *GNLY, HOXB7, MRPL33, PRDM16, TCF7*, and *ZNF26* were determined to be potential diagnostic and therapeutic biomarkers for PitNETs.

**Conclusion:**

This study illustrated the significant influence of eRNAs on the occurrence and development of PitNETs. By constructing the co-expression regulation network, *GNLY, HOXB6, MRPL33, PRDM16, TCF7*, and *ZNF26* were identified as relatively significant DEEs which were considered as the novel biomarkers of diagnosis and treatment of PitNETs. This study demonstrated the roles of eRNAs in the occurrence and development of PitNETs and revealed that ciclopirox was a potential therapeutic for pituitary adenomas.

## 1 Background

Pituitary neuroendocrine tumors (PitNETs), which are a common type of adenoma, originate from the pituitary gland (1). Epidemiological studies have indicated that PitNETs affect more than 5% of the global population. Functional PitNETs secrete specific hormones, including prolactin, growth hormone, and cortisone hormones, contributing to the development of severe endocrine disorders and fatal outcomes (2). In contrast, non-functional PitNETs invade or exert pressure on neighboring healthy tissues (3). The major aims of PitNET treatment are the suppression of excessive hormonal secretion and tumor size to improve clinical symptoms (4). The frontline treatments for PitNETs include pituitary surgery, pharmacological treatment for specific subtypes (including somatostatin analogs and dopamine agonists), systemic chemotherapy, and/or radiotherapy (3). Patients with PitNETs may exhibit hypopituitarism, cerebrospinal fluid leaks, and diabetes insipidus. Approximately 10% of patients with PitNETs exhibit tumor recurrence within 10 years of surgery (5). Although radiation therapy inhibits tumor growth for several years, adenoma-associated hormone hypersecretion may persist for some years. Most patients experience pituitary failure within 10 years of radiotherapy and require lifelong hormone replacement therapy (6). Currently, the efficacies and adverse effects of various therapeutic approaches vary. Thus, there is an urgent need to identify novel therapeutic biomarkers for PitNETs to develop efficient therapies.

Enhancers are sequences located around a gene, especially upstream of the transcription start site, and serve as a binding site for transcription factors (TFs) to regulate gene expression (7). Enhancer RNAs (eRNAs), which are a class of non-coding RNAs transcribed from the enhancer regions of the genome (8), promote target gene expression, facilitate interactions with TFs, and regulate the epigenome (9). Recent studies have demonstrated the role of eRNAs in gene regulation in both cancerous and non-cancerous cells (10). Some tumor suppressor genes, such as *TP53* can regulate tumor cell behavior through the production of eRNAs (11). Additionally, eRNAs can directly regulate tumor development under specific conditions (12). eRNAs exhibit tissue-specific and individual-specific expression patterns (13). Thus, eRNAs are potential diagnostic and prognostic biomarkers, as well as novel therapeutic targets, for cancer (14).

The role of eRNAs in the development of PitNETs has not been completely elucidated. This study comprehensively analyzed data from public databases to investigate the differential expression of eRNAs between PitNET and non-tumor samples. To investigate the correlation between genomic eRNAs and immune cell infiltration, the correlation of eRNAs with the abundance of immune cells (determined using CIBERSORT) and immune-related pathways (identified using single-sample Gene Set Enrichment Analysis (ssGSEA) algorithms) was examined. Moreover, a correlation analysis was performed by integrating TFs, eRNAs and their target genes, the abundance of immune cells, the enrichment score of immune-related gene sets, and cancer hallmark gene sets (signaling pathways were determined using Gene Set Variation Analysis, GSVA). Based on the correlation analysis, a co-expression regulatory network was constructed to explore the underlying regulatory mechanisms of eRNAs, which comprised key components, including target genes, differentially expressed TFs, immune cells, and gene sets. Additionally, Connectivity Map (CMap) analysis was performed to identify potential small-molecule inhibitors with anti-PitNET properties. To validate these findings, sequencing data from the public database (assay for transposase-accessible chromatin with sequencing (ATAC-Seq) and chromatin immunoprecipitation sequencing (ChIP-Seq) data) and experimental approaches were utilized for external validation.

## 2 Methods

### 2.1 Primary data collection

In order to calculate the expression of differentially expressed enhancer RNAs (DEEs) and target genes in patients with pituitary neuroendocrine tumors (PitNETs), we analyzed gene expression profiles of 134 PitNET tissue samples. These profiles were obtained from the supplementary appendix of an article published in Cancer Cell (15). To provide control samples, we used RNA-sequencing (RNA-seq) data from 107 normal pituitary tissues extracted from the Genotype-Tissue Expression (GTEx) database (https://commonfund.nih.gov/gtex). Moreover, the results of gene sets (including 50 hallmarks of cancer gene sets and 29 immune gene sets) and 318 PitNETs-related transcription factors (TFs) were obtained separately from Molecular Signatures Database (MSigDB) v7.1 (https://www.gsea-msigdb.org/gsea/msigdb/index.jsp) (16), and the Cistrome database (http://cistrome.org) (17). With the annotation of Ensemble ID, the standardized gene expression profiling of eRNAs in PitNETs was acquired from the enhancer RNA in cancers (eRic) database (https://hanlab.uth.edu/eRic/) (18). Afterward, in terms of the position in hg38 genome, we used the ChIPseeker package to identify the formal gene mark of each eRNA (19).

### 2.2 Analysis of gene expression, functional enrichment, and clinical relevance

At the beginning of our statistical analysis, edgeR and limma algorithm (20, 21) were used respectively to identify differentially expressed genes (DEGs) based on specific screening criteria (|log_2_ FC|> 1.0 (FC > 2.0), FDR < 0.05|) in the whole transcriptome and eRNAs. Furthermore, the cellular components (CCs), molecular functions (MFs), biological processes (BPs), and KEGG pathways of DEGs enrichment were illuminated by functional enrichment analysis (Gene Oncology (GO) and Kyoto Encyclopedia of Genes and Genomes (KEGG) items) (22). As for the analysis of clinical relevance, staging and cell type, which was related to prognosis, were considered as screening factors to identify clinical-related differentially expressed eRNAs (DEEs).

### 2.3 Identification of immune cells abundance, hallmarks of cancer-related gene sets, and differentially expressed target genes of eRNAs

To construct the eRNA regulatory network of PitNETs, we employed the CIBERSORT algorithm to calculate the proportions of 22 different types of immune cells across all samples (23). Furthermore, the infiltration of immune cells in the PitNETs samples and normal cases differed significantly, as determined by the Kruskal-Wallis and Mann-Whitney tests. Then, 29 immune gene sets and 50 hallmarks of cancer gene sets were quantified based on ssGSEA algorithm *via* GSVA R package (24). The edgeR package (21) was used to identify differential hallmarks of cancer gene sets or immune cells between PitNETs samples and the normal pituitary samples. The significant level was set to FDR < 0.05.

### 2.4 Construction of the regulation network and connectivity map analysis

To explore the role of eRNA and its related factors in the development of pituitary tumors, we constructed a specific co-expression regulatory network that included clinical-related DEEs, target genes of eRNAs, hallmarks of cancer gene sets, differentially expressed transcription factors (TFs), immune cells, and immune gene sets. Pearson correlation analysis was utilized to identify interaction pairs between these components (|correlation coefficient| > 0.70 and P < 0.05 for hallmarks of cancer gene sets and clinical-related DEEs; |correlation coefficient| > 0.90 and P < 0.05 for differentially expressed TFs and clinical-related DEEs), which were deemed significant elements of the network. Additionally, interaction pairs between target genes and clinical-related DEEs, immune cells and clinical-related DEEs, and immune gene sets and clinical-related DEEs (|correlation coefficient| > 0.85 and P < 0.05, |correlation coefficient| > 0.60 and P < 0.05, and |correlation coefficient| > 0.80 and P < 0.05, respectively) were included. We then utilized the Connectivity Map analysis (CMap) to identify potential small molecules that target significant components in the co-expression regulatory network of the pituitary adenomas, based on clinical-related DEEs, target genes of eRNAs, hallmarks of cancer gene sets, differentially expressed TFs, immune cells, and immune gene sets (25, 26). Finally, the heatmap showed several small molecule inhibitors that were shown to be effective against numerous types of cancers.

### 2.5 Validation

Since our data was not extracted from TCGA, only String (27) were utilized to validate the key DEEs in order to reduce biases and verify the accuracy of the study. Additionally, the data of ATAC-seq was extracted from The Cancer Genome Atlas (TCGA) (https://tcga-data.nci.nih.gov) to verify the accessibility of the key DEEs (28). Moreover, the binding level between *PRDM16* and ELK4 was explored by using the CHIP-seq downloaded from the Cistrome database (http://cistrome.org/) (29–31). The information of clinical data, target genes, and drugs was downloaded from the eRic database (https://hanlab.uth.edu/eRic/) (18). Lastly, four small molecule inhibitors (ciclopirox, bepridil, clomipramine, and alexidine) were tested using the CellTiter-Glo luminescent cell viability assay (Promega, WI, USA) on GH3 cell line as per the manufacturer’s instructions. Among these compounds, ciclopirox was identified as a potential treatment for pituitary adenomas *in vitro* and *in vivo*.

### 2.6 Animal experiments

Athymic mice were purchased from Charles River (Shanghai, China) and kept in a laboratory with SPF-class conditions. For the subcutaneous transplantation animal studies, 1×10^6^ GH3 cells were suspended in 100 μl PBS and bilaterally injected into the subcutaneous region of an athymic mouse. On the 30th day, the tumor volume was assessed using a Vernier caliper and calculated using the formula V= 1/2×L×W^2^. The athymic mice were divided into two groups: the experimental group and the control group. The experimental group received intraperitoneal administration of ciclopirox at a dosage of 10 mg/kg, repeated at 48-hour intervals (32), in a volume of 100 μl PBS. All mice were euthanized, and tumor samples were collected for subsequent analysis.

### 2.7 Immunohistochemistry

Tissue samples were fixed with 4% formalin and embedded in paraffin. The slices were cut to 5 μm thickness. IHC was performed by blocking the rehydrated tissue sections using bovine serum overnight at 4°C, and subsequently applying the Anti-Ki67 antibody (Abcam, Cat. no. ab15580) and Anti-PCNA antibody (Abcam, Cat. no. ab92729). The sections were washed and then subjected to incubation with biotinylated anti-mouse IgG or biotinylated anti-rabbit IgG (Vector Laboratories, CA, USA). The ABC method (Vector Laboratories, CA, USA) was employed along with 3,3’- diaminobenzidine (Dojindo Laboratories, Kumamoto, Japan) as a substrate to detect the staining. The sections were visualized using an AX-80 microscope (Olympus, Tokyo, Japan), and Image J software (http://imagej.nih.gov/ij/) was employed for image analysis and quantification of positive expression. Statistical analysis was conducted using One-Way ANOVA.

### 2.8 Cell proliferation assays

CellTiter-Glo luminescent cell viability assay (Promega, WI, USA), a test system to detect the amount of present ATP proportional to the cell number, was employed to estimate the cell proliferation. GH3 cells were inoculated and treated with cyclopentanol (MCE, CAS No. 29342-05-0, 0-2 μm) for 48 hours (n=3), bepridil (MCE, CAS No. 64706-54-3, 0-100 μM) for 48 hours (n=3), chlorpromazine (MCE, CAS No. 303-49-1, 0-100 μM) for 48 hours (n=3), and alexidine (MCE, CAS No. 22573-93-9, 0-100 μM) for 48 hours (n=3), in 15% HS-2.5% FBS-F12K culture medium. All drugs were dissolved in DMSO and an equal amount of DMSO was added to the control group. Then, the CellTiter-Glo reagent was added into each well at the equivalent volume of cell culture medium in the well. Then, the contents were mixed vigorously for 5 min to induce cell lysis, and the plate was incubated at room temperature for an additional 25 min to stabilize the luminescent signal. Afterwards, 200 µL supernatants were transferred in technical replicates into the 96-well opaque-walled plate and the luminescence was measured. Analysis of the luminescence signal was performed using GraphPad Prism 9.0, with data being normalized to the control group, and p-values calculated through one-way ANOVA. At least three independent experiments were conducted to obtain the results.

### 2.9 Statistics analysis

During the process of study, the R software (https://www.r-project.org/; version 3.6.1; Institute for Statistics and Mathematics, Vienna, Austria) was used to conduct all statistical analyses. P-values were adjusted using FDR and the significant level was set to P-value < 0.05 or FDR < 0.05 (for multiple testing).

## 3 Results

### 3.1 Analysis of expression, functional enrichment, and clinical relevance of enhancer RNAs in PitNETs

**Figure 1:**
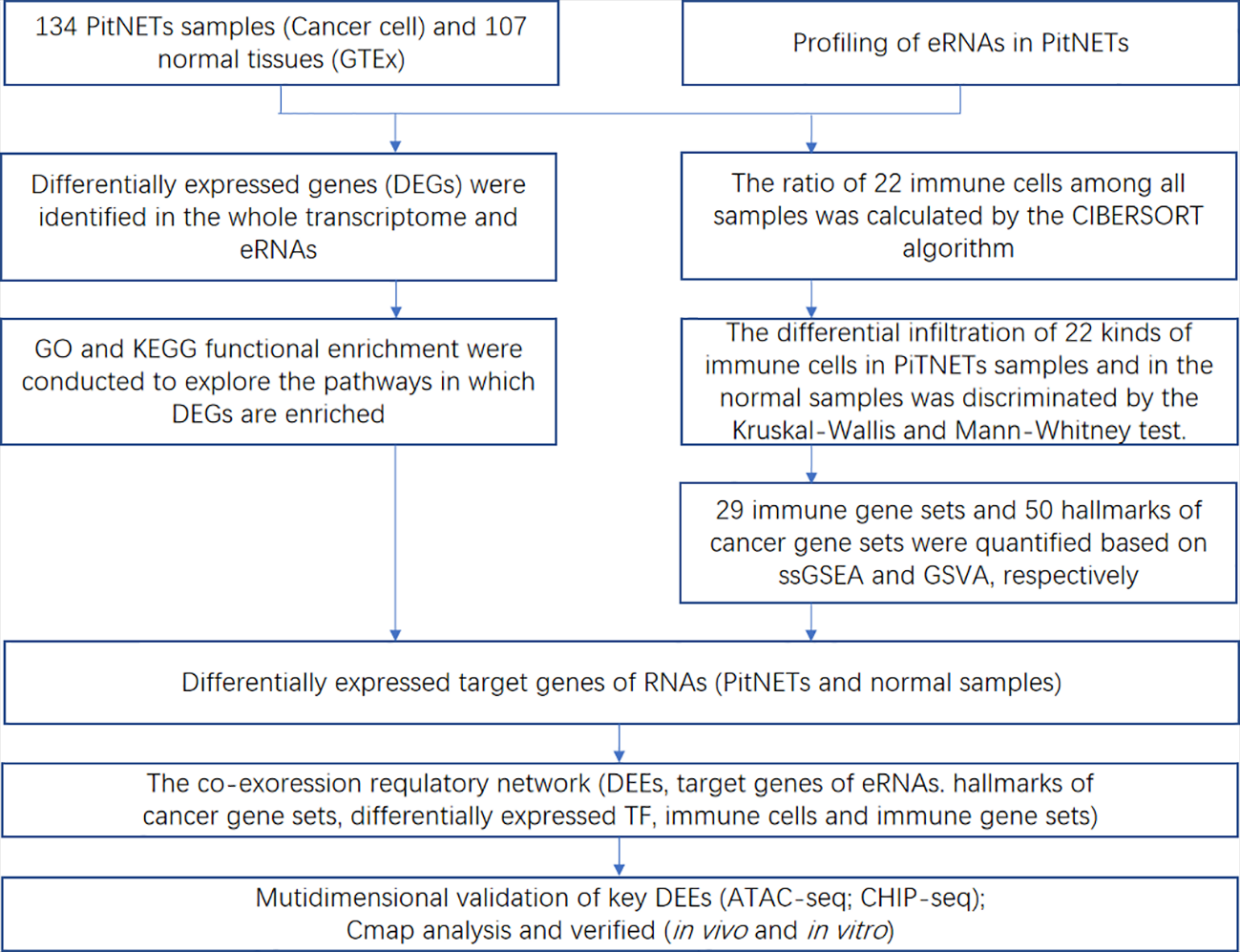
The flowchart showing each procedure of this study in sequence.

**Figure 2:**
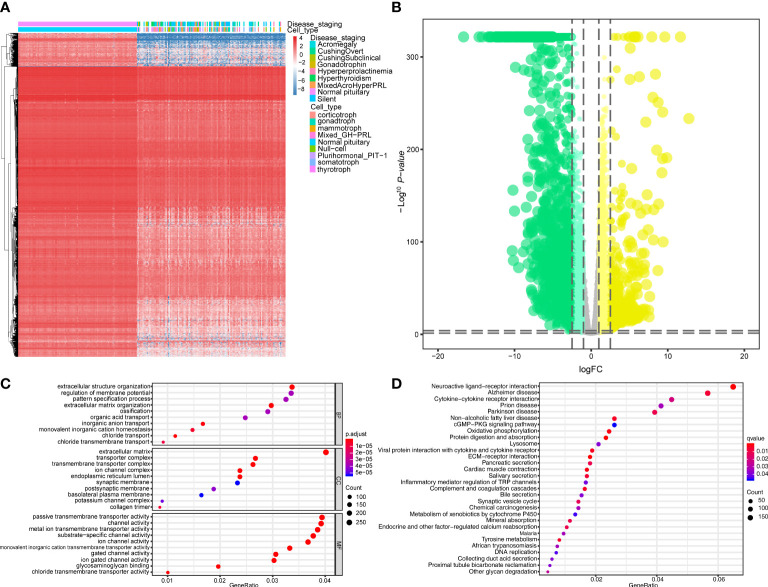
Analysis of differential expression and functional enrichment. **(A, B)** Differentially expressed genes (DEGs) between PitNETs and normal samples were shown in the heatmap and violin plot. **(C, D)** The results of GO and KEGG functional enrichment analysis were displayed in the dotplots.

**Figure 3:**
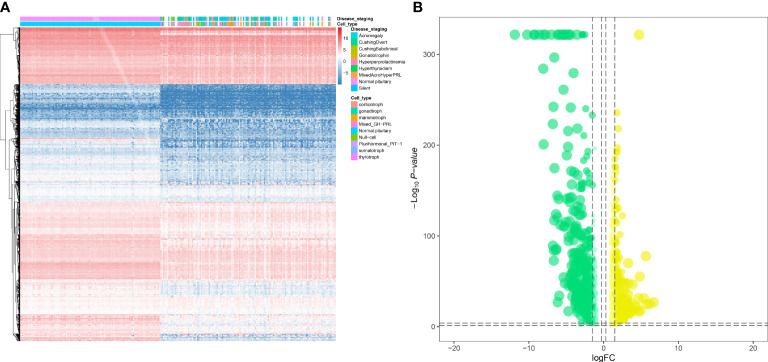
Identification of differentially expressed eRNAs (DEEs). The heatmap **(A)** and the violin plot **(B)** showing the key DEEs between PitNETs and normal samples.

The workflow of this study is shown in **Figure 1**. This study identified 7,267 differentially expressed genes (DEGs) (3,413 upregulated genes and 3,854 downregulated genes) between 134 PitNET and 107 normal pituitary samples. The DEGs were selected based on the following criteria to ensure robust and significant differences in gene expression between the two sample groups: false discovery rate (FDR) value < 0.05; |log2 fold-change (FC)| > 1.0 (**Figures 2A, B**). Kyoto Encyclopedia of Genes and Genomes and Gene Ontology functional enrichment analyses revealed that the most significantly enriched signaling pathway and molecular function were neuroactive ligand-receptor interaction and extracellular matrix, respectively (**Figures 2C, D**). The ChIPseeker package was used to identify the official gene symbol of each eRNA according to its location in the hg38 genome. Based on the staging and cell type, 1128 clinically relevant differentially expressed eRNAs (DEEs) (494 upregulated eRNAs and 634 downregulated eRNAs) were identified between PitNET and healthy pituitary samples (**Figures 3A, B**).

### 3.2 Identification of immune cells and immune gene sets in PitNETs

**Figure 4:**
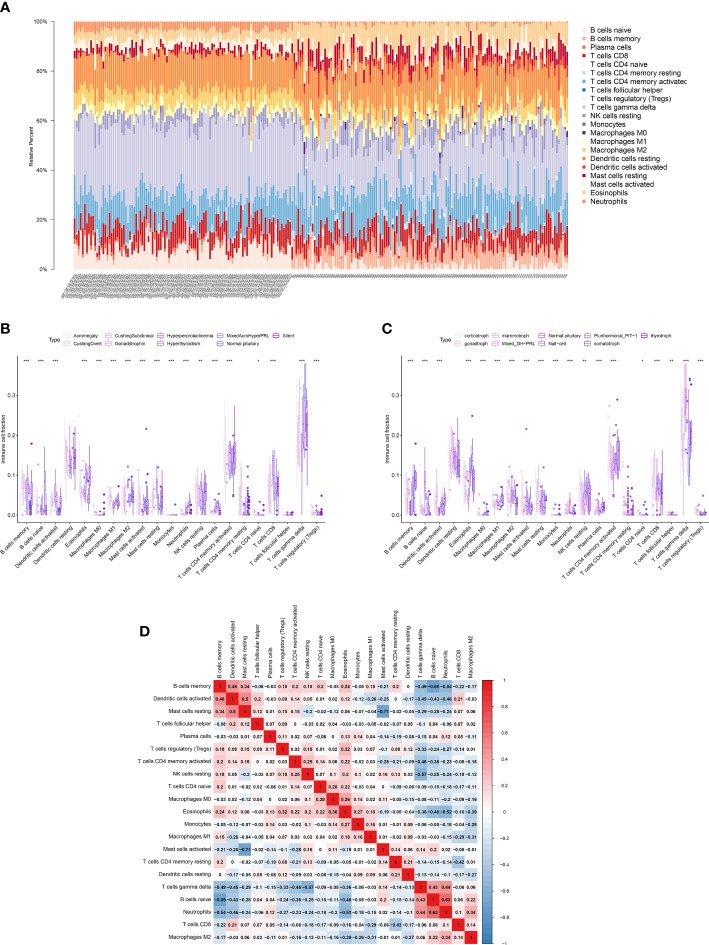
Quantification of immune cells. **(A)** The proportions of 22 types of immune cells in each PitNETs was shown in the bar plot. **(B, C)** The plots showing the differential expression level of the key DEEs on disease staging and cell types. **(D)** The heatmap showing the co-expression of 22 kinds of immune cells. *p<0.05; **p<0.01; ***p<0.001.

The CIBERSORT algorithm was used to calculate the proportion of 22 immune cell types in the healthy pituitary and PitNET samples. The results are presented as a bar plot in **Figure 4A**. Next, the differential abundances of 22 immune cell types between the PitNETs and healthy pituitary samples were determined using the Kruskal-Wallis and Mann-Whitney tests. The differentially abundant immune cell types are shown in **Figures 4B, C**. The nonparametric test results revealed a significant correlation between immune cells/pathways and PitNETs. **Figure 4D** shows the co-expression coefficient between each pair of immune cells.

### 3.3 Differential expression analysis of TFs and cancer hallmark gene sets and functional enrichment of PitNETs gene sets

**Figure 5:**
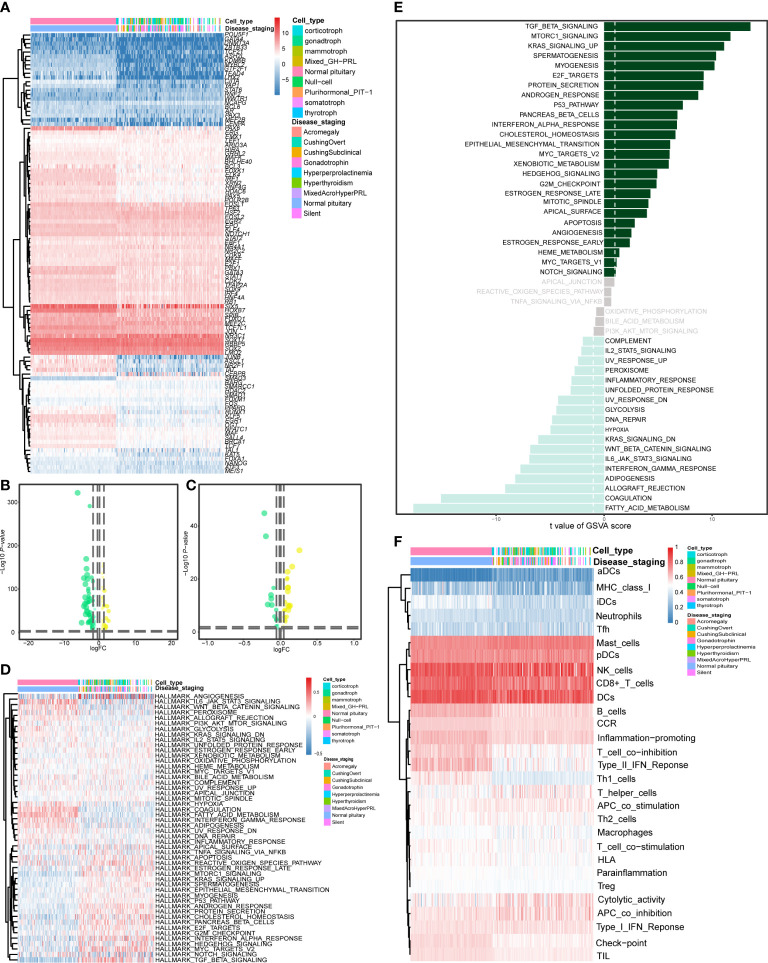
Differentially expressed analysis of TFs, hallmarks of cancer genes and immune gene sets. The heatmap **(A)** and the violin plot **(B)** showing the differentially expressed TFs between the PitNETs and normal samples. The heatmap **(C)** and the violin plot **(D)** showing the differentially expressed hallmarks of cancer genes between the PitNETs and normal samples. **(E)** The bar chart showing the key hallmarks of cancer (including 26 upregulated and 28 down-regulated hallmarks). **(F)** The heatmap illustrating the differential expression of 29 immune gene sets between the PitNETs and normal samples.

**Figures 5A–D** shows 105 differentially expressed TFs and 50 differentially expressed cancer hallmark gene sets between 134 PitNETs and 107 healthy samples. Additionally, **Figure 5E** shows a t-test bar graph comprising 26 upregulated and 28 downregulated cancer hallmark gene sets. The top 2 upregulated cancer hallmarks were TGF-β signaling (t = −13.548, P < 0.001) and mTORC1 signaling (t = −11.702, P < 0.001), whereas the most downregulated cancer hallmark was fatty acid metabolism (t = 17.671, P < 0.001). This result was in contrast to that of most of the 44 significant hallmarks. **Figure 5F** shows the results of ssGSEA for the differential expression of 29 immune gene sets between PitNETs and healthy samples (FDR < 0.05). As shown in **Figures S1A, B**, 937 differentially expressed target genes of eRNAs are represented as a heatmap and a violin plot.

### 3.4 Co-expression regulatory network and CMap analysis

**Figure 6:**
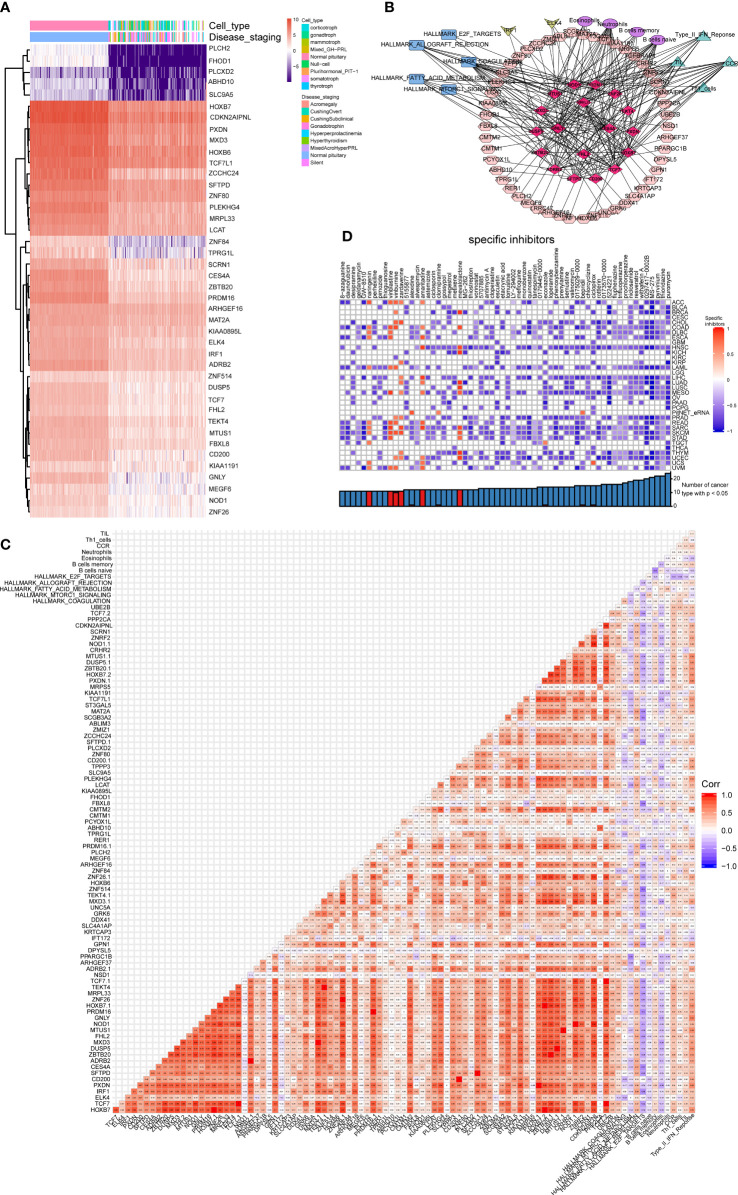
Construction of the regulated network. **(A)** The heatmap showing the key DEGs in the network between the PitNETs and normal samples. **(B)** The co-expression regulated network. Red rhombus represented 18 significant DEEs, pink octagon represented 50 target genes of eRNAs, blue rectangle represented 5 hallmarks of cancer gene sets, yellow quadrilateral represented 2 differentially expressed TF, purple circle represented 4 types of immune cells and green triangle represented 4 kinds of immune gene sets. **(C)** The complicated co-expression coefficient of such several components of the network was shown in the heatmap. **(D)** Effective small molecular inhibitors were illustrated in the heatmap.

Summarizing the above analysis revealed 42 DEGs between 134 PitNETs and 107 healthy samples, which were represented in a heatmap (**Figure 6A**). Based on the criteria mentioned in the method, a co-expression regulatory network comprising 18 clinically correlated DEEs, 50 target genes of eRNAs, 5 cancer hallmark gene sets, 2 differentially expressed TFs, 4 types of immune cells, and 4 immune gene sets were established (**Figure 6B**). Additionally, the heatmap illustrated the complex co-expression relationships among several components of the network (**Figure 6C**). CMap analysis was performed to distinguish specific small-molecule inhibitors of DEGs in the network. The data included clinically related DEEs, differentially expressed TFs, different target genes of eRNAs, cancer hallmark gene sets, various types of immune cells, and immune gene sets. As shown in **Figure 6D**, ciclopirox (specificity = 0.133, P < 0.01), bepridil (specificity = 0.094, P < 0.01), clomipramine (specificity = 0.171, P < 0.01), and alexidine (specificity = 0.010, P < 0.01) were the most effective molecular inhibitors for key DEEs.

### 3.5 External database validation

The six most significant DEEs (three upregulated eRNAs and three downregulated eRNAs) were identified from clinical correlation analysis (*GNLY*, *HOXB7*, *MRPL33*, *PRDM16*, *TCF7*, and *ZNF26*). The validation was conducted using String databases (**Figure S2**). The chromatin accessibility of the six key DEEs was examined. The peaks indicated the regions of the chromatin (**Figures S3A–F**). ChIP-seq analysis revealed that the correlation coefficient of the interaction pairs of *PRDM16* (eRNA) and ELK4 (TF) was the highest (cor = 0.909) (**Figure S4**). Additionally, *GNLY*, *HOXB7*, *MRPL33*, and *PRDM16* were upregulated in bladder urothelial carcinoma, colon adenocarcinoma, lung adenocarcinoma, and rectum adenocarcinoma based on the results of eRic validation (**Figure S5**; **Table S1**).

### 3.6 *In vitro* and *in vivo* validation

**Figure 7:**
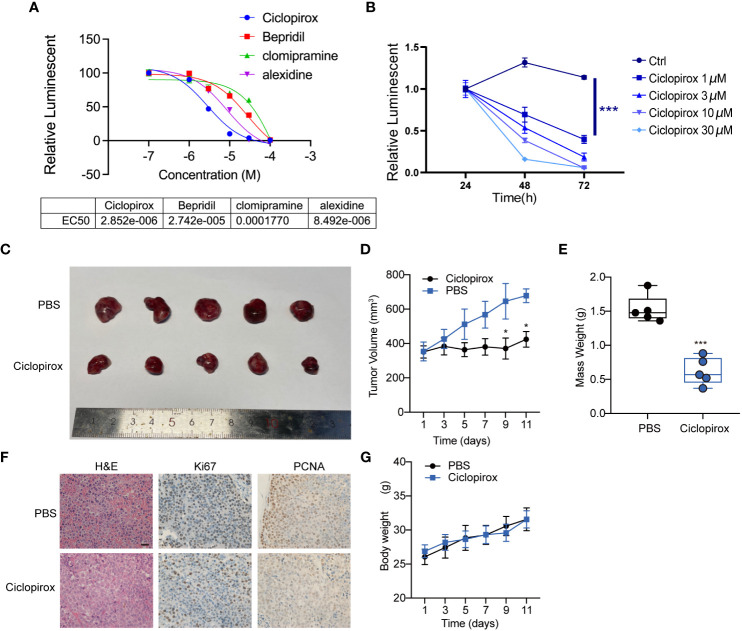
The effect of four molecular inhibitors on GH3 cell line *in vitro* **(A)** and the effect of ciclopirox on primary GH-secreting pituitary adenoma cells *in vitro* **(B)**. Cell survival was determined by CellTiter-Glo luminescent cell viability assay. The effect of ciclopirox *in vivo*. Representative images **(C)**, tumor growth curve **(D)**, mass weight **(E)**, representative images of HE, ki67 and PCNA staining on tumor section of mice indicated in **(F)**, and body weight **(G)** of tumors derived from athymic tumor-bearing mice treated with PBS or ciclopirox. *p<0.05; ***p<0.001.

To assess the therapeutic potential of the screened drugs for pituitary tumors, the growth-inhibitory effects of four drugs against the GH3 cell line were examined. Among the tested small-molecular inhibitors, ciclopirox exhibited the highest efficacy (half-maximal effective concentration = 2.85 μM) against the GH3 cell line (**Figure 7A**). Subsequently, primary growth hormone (GH)-secreting adenoma cells were seeded in a 96-well plate and treated with different concentrations of ciclopirox. Ciclopirox concentration-dependently decreased the viability of primary GH-secreting pituitary adenoma cells (**Figure 7B**). To examine the effect of ciclopirox on PitNETs *in vivo*, a athymic mouse model bearing subcutaneous transplants of GH3 cell line-derived tumors was established. To establish the tumor xenograft model, athymic mice were subcutaneously injected with GH3 tumor cells on both sides of the skin. On day 30 post-injection, the tumor volume was approximately 300 mm^3^. The athymic mice were screened and classified based on the mean tumor size for further experiments. To determine the treatment effectiveness, the mice were intraperitoneally administered with ciclopirox or phosphate-buffered saline every other day, and tumor size was measured regularly. On day 41, the mice were euthanized, and the tissue samples were collected for further analysis. Ciclopirox did not exert adverse effects on mouse health. At the end of the experimental period, ciclopirox significantly inhibited the growth of subcutaneous tumors in athymic mice. The average tumor volumes in the experimental group (382.95 mm^3^) were significantly lower than those in the control group (629.87 mm^3^) (p < 0.05) (**Figures 7C–E**). Consistently, immunohistochemical staining revealed that the levels of cellular proliferation markers (MKI67 and PCNA) were downregulated in the ciclopirox-treated cells, as well as in subcutaneously transplanted tumors in athymic mice (**Figure 7F**). However, the bodyweight of mice was not significantly different between the two groups (**Figure 7G**). This indicates that ciclopirox did not affect the health of the mice at the tested dose. Thus, ciclopirox can suppress the proliferation of pituitary tumor cell lines *in vitro* and *in vivo*, as well as inhibit the growth of GH-secreting tumors.

## 4 Discussion

PitNETs are the most common pituitary disorder (33), accounting for 10%–15% of all intracranial tumor cases (34). Suspected PitNET cases are diagnosed based on the symptoms. Moreover, the diagnostic methods recommended for PitNETs are based on expert opinions and observational research, which are in turn based on prior clinical experience and research findings (35–37). The currently used therapeutic approaches for PitNETs are associated with adverse effects and/or delay disease onset. Furthermore, clinical trials comparing early therapy with conservative treatment have not been performed. Thus, the intervals for management and monitoring rely on expert opinions (38). Consequently, there is an urgent need to identify novel potential biomarkers to develop diagnostic and therapeutic strategies for PitNETs. Additionally, previous bioinformatics analysis findings on pituitary adenomas have piqued the interest of the scientific community. Currently, the main research focus of pituitary adenoma studies is proteogenomics and microRNAs. Meanwhile, limited studies have examined the role of circular RNAs. However, the bioinformatics analysis of eRNAs has not been previously performed (38–40). A recent study reported that dysfunctional enhancers are associated with the pathogenesis of various human cancers (41). Based on the expression patterns of eRNAs in human neoplasm cells, the regulation of eRNA expression is potentially a novel therapeutic approach for tumors (42–46). The mechanism of action of eRNAs in the development of PitNETs has not been elucidated.

In this study, 1128 DEEs (494 upregulated eRNAs and 634 downregulated eRNAs) were identified between 134 PitNETs and 107 healthy pituitary samples. The proportion of immune cells in the tumor microenvironment is a crucial biomarker for predicting therapy response and identifying patients who will benefit from immunotherapy. The CIBERSORT, ssGSEA, and GSVA algorithms were used to identify the proportion of 22 different types of immune cells, immune gene sets, and cancer hallmark gene sets, respectively. The final co-expression regulatory network was constructed using 18 DEGs, 50 target genes of eRNAs, five cancer hallmark gene sets, two differentially expressed TFs, four types of immune cells, and four immune gene sets. CMap analysis revealed that ciclopirox, bepridil, clomipramine, and alexidine are effective therapeutics for PitNETs. The top three upregulated and downregulated eRNAs (*GNLY*, *HOXB7*, *MRPL33*, *PRDM16*, *TCF7*, and *ZNF26*) were validated using ATAC-seq and online datasets.

In the human genome, *GNLY* encodes granulysin, a cytolytic granule protein secreted from natural killer cells and cytotoxic T lymphocytes (47). *GNLY* exerts tumor suppressor effects. The serum concentrations of granulysin determine the immune response in patients with cancer (48, 49). Granulysin exhibits pro-inflammatory, cytotoxic, antitumor, and antimicrobial properties (50, 51). Previous studies have suggested that serum granulysin levels are biomarkers in patients with benign tumors and malignant diseases. However, limited studies have examined serum granulysin concentrations in patients with PitNETs (52). *HOXB7*, which belongs to class I homeobox genes, is involved in tumor progression and tumorigenesis. The dysregulation of *HOXB7* is reported to induce multiple tumors (53). For example, *HOXB7* is dysregulated in tumors, such as colorectal and pancreatic cancers and is associated with differentiation and tumor grade (54). Recent studies have confirmed that *HOXB7* is associated with tumor cell invasiveness and proliferation and clinicopathological features (53). *MRPL33* is one of the 50 genes encoding the mitoribosome subunit. *MRPL33*-L and *MRPL33*-S are the transcriptional variants of *MRPL33* and are generated *via* alternative splicing of exon 3 (55). The two variants exert contrasting effects on the apoptosis and proliferation of cancer cells (55). However, the effects of the two *MRPL33* isoforms on cancer have not been completely elucidated. Further studies on the mechanisms and functions of *MRPL33* may contribute to the development of personalized and effective treatment strategies (56). *PRDM16*, a TF, promotes mitochondrial respiration and oxidative metabolism in brown fat cells (57). Additionally, *PRDM16* enhances the transcriptional activity of the peroxisome proliferator-activated receptors, promoting the transcription of metabolic genes in fat cells and regulating the activity of some stem cell populations (58). A recent study reported that *PRDM16* inhibits the development of tumors (59). The loss of PRDM family members, including *PRDM16*, in kidney and lung cancer suggested that *PRDM16* is involved in the pathogenesis of multiple cancers. *TCF7* (also known as *TCF-1*), which belongs to the TCF family, comprises a β-catenin-binding domain and a high-mobility group DNA-binding domain. The β-catenin-binding domain can interact with nuclear β-catenin, while the high-mobility group DNA-binding domain can identify DNA and switch transcription activities toward Wnt signaling (60). *TCF7* upregulation was recently reported to be related to the development and poor prognosis of multiple tumors. Additionally, some miRNAs suppress tumor carcinogenesis and progression by downregulating the expression of *TCF7* (61). *ZNF26* regulates herpes simplex virus 1 infection response-related and gene expression-related pathways. *ZNF26*8 is a paralog of *ZNF26* (https://www.genecards.org/cgi-bin/carddisp.pl?gene=ZNF26). In this study, the aberrant expression of *ZNF26* was reported to promote the occurrence of PitNETs.

Ciclopirox, a broad-spectrum fungicide, has been used to treat pathogenic dermatophytes, yeasts, and *Malassezia furfur* (62, 63). Recent preclinical and clinical studies have demonstrated that ciclopirox exhibits anti-cancer activities (63) and exerts therapeutic effects on diabetes (64, 65), cardiovascular disorders (66), and acquired immune deficiency syndrome (67). Ciclopirox exerts anti-cancer effects *in vitro* by inhibiting cell proliferation, angiogenesis, and lymphangiogenesis, inducing apoptosis, and suppressing cell migration and invasion (68). *In vivo* studies have reported that the growth rate of human leukemia and human breast xenograft in mice administered with ciclopirox (25 mg/kg bodyweight) *via* oral gavage is suppressed by up to 65% and 75%, respectively, when compared with that in control mice (69, 70). Additionally, ciclopirox (20 mg/kg bodyweight) potently inhibited the growth of human colon tumors in mice (71). Furthermore, clinical studies have indicated that the oral administration of ciclopirox (40 mg/m^2^) for five days resulted in disease stabilization and/or hematological improvement in 2/3 patients with advanced hematological malignancies (NCT00990587) (72). The anti-cancer mechanisms of ciclopirox are complex and include the inhibition of Wnt/β-catenin, histone demethylases/Myc, VEGFR-3/ERK1/2, cyclin-dependent kinases, and mTORC1 and the induction of reactive oxygen species or activation of ATR/Chk (68). In this study, ciclopirox (10 mg/kg bodyweight) effectively inhibited pituitary tumor cell proliferation *in vitro*. Consistently, the intraperitoneal injection of ciclopirox significantly suppressed tumor size in a mouse model. The toxicological and pharmacological profiles indicated that the systemic administration of ciclopirox, especially ciclopirox-POM (ciclopirox prodrug), is feasible and safe (73). Preclinical and clinical data suggest that ciclopirox can be repositioned as an anti-PitNET drug.

This study examined the novel roles of eRNAs in PitNETs. However, this study has several limitations. The Cancer Genome Atlas database did not provide relevant data on pituitary tumors. Hence, the tumor data from other sources and the non-cancerous tissue data from the Genotype-Tissue Expression database were used for analysis. The PitNET data were extracted from the article published in Cancer Cell (15). These data may be limited and associated with statistical bias. Therefore, external data should be used to verify the findings of this study. Additionally, the findings of this study may be associated with geographical bias as the analyzed samples were only from the American population. Furthermore, this study only focused on investigating the role of eRNAs in PitNETs. Future studies must examine the role of eRNAs in different PitNET subtypes. Finally, validation was limited to *in vitro* experiments involving the GH3 cell line and a single GH-secreting primary tumor owing to the limited availability of primary cells. Therefore, additional cellular, animal, and clinical experiments must be performed with various types of PitNETs to accurately validate the bioinformatics analysis results of this study.

## 5 Conclusions

This study demonstrated the roles of eRNAs in the development and progression of PitNETs. Furthermore, a co-expression regulatory network was constructed, which revealed the following six DEEs: *GNLY*, *HOXB7*, *MRPL33*, *PRDM16*, *TCF7*, and *ZNF26*. These DEEs are potential diagnostic and therapeutic biomarkers for PitNETs. Additionally, *in vivo* experiments demonstrated that ciclopirox exerts therapeutic effects on pituitary adenomas.

## Data availability statement

The datasets presented in this study can be found in online repositories. The names of the repository/repositories and accession number(s) can be found in the article/**Supplementary Material**.

## Ethics statement

The animal study was reviewed and approved by Institutional Animal Care and Use Committee at Shanghai Jiao Tong University School of Medicine.

## Author contributions

SL, YD, LX, and ZW designed the study and carried out the data analyses. LW, CW, YW, NH, and TZ interpreted the results. LW and CW drafted the manuscript. YD, LX, SL and ZW revised the manuscript. All authors contributed to the article and approved the submitted version.

## Supplementary material

Supplementary Figure 1 The heatmap **(A)** and the violin plot **(B)** showing the differentially expressed target genes of key eRNAs between the PitNETs and normal samples.

Supplementary Figure 2 The result of validation from String.

Supplementary Figure 3 Results of ATAC-seq analysis. The accessibility on chromatin of **(A)** *GNLY*, **(B)** *HOXB7*, **(C)** *MRPL33*, **(D)** *TCF7* and **(E)** *ZNF26* was verified by ATAC-seq analysis.

Supplementary Figure 4 The binding level between *PRDM16* (eRNA) and ELK4 (TF) in response to the CHIP-seq analysis.

Supplementary Figure 5 The expression level of **(A)** *GNLY*, **(B)** *HOXB7*, **(C)** *MRPL33* and **(D)** *PRDM16* between the PitNETs and normal samples based on the eRic database. Kaplan Meier curves (KM_CURVE) of overall survival of **(E)** *GNLY*, **(F)** *HOXB7*, **(G)** *MRPL33* and **(H)** *PRDM16* based on the eRic database.
