# Supplementary figures and images for: Identification of the enhancer RNAs related to tumorgenesis of pituitary neuroendocrine tumors
Source: Front Endocrinol (Lausanne). 2023 Jul 18;14:1149997. doi: 10.3389/fendo.2023.1149997 (PMC10393250; doi:10.3389/fendo.2023.1149997)

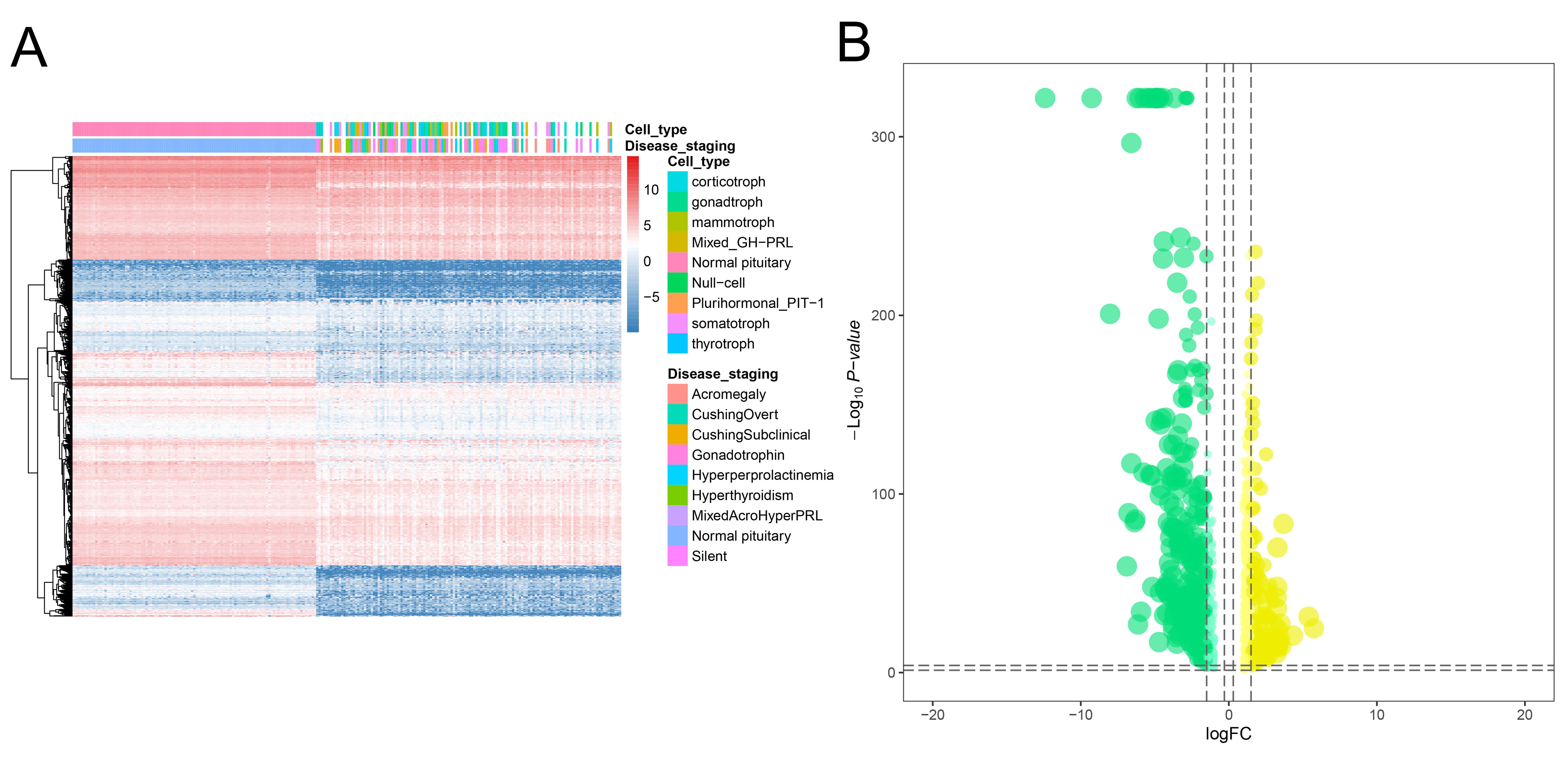

Supplement: Supplementary Figure 1 — The heatmap (A) and the violin plot (B) showing the differentially expressed target genes of key eRNAs between the PitNETs and normal samples. [file Image_1.tif]

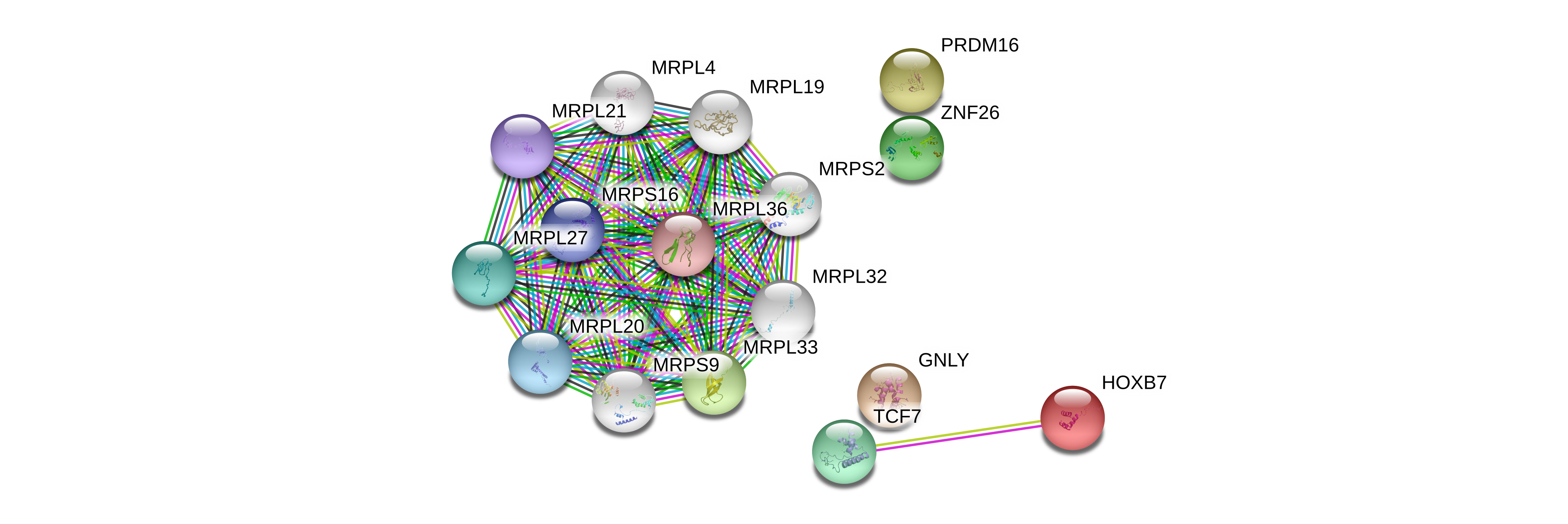

Supplement: Supplementary Figure 2 — The result of validation from String. [file Image_2.tif]

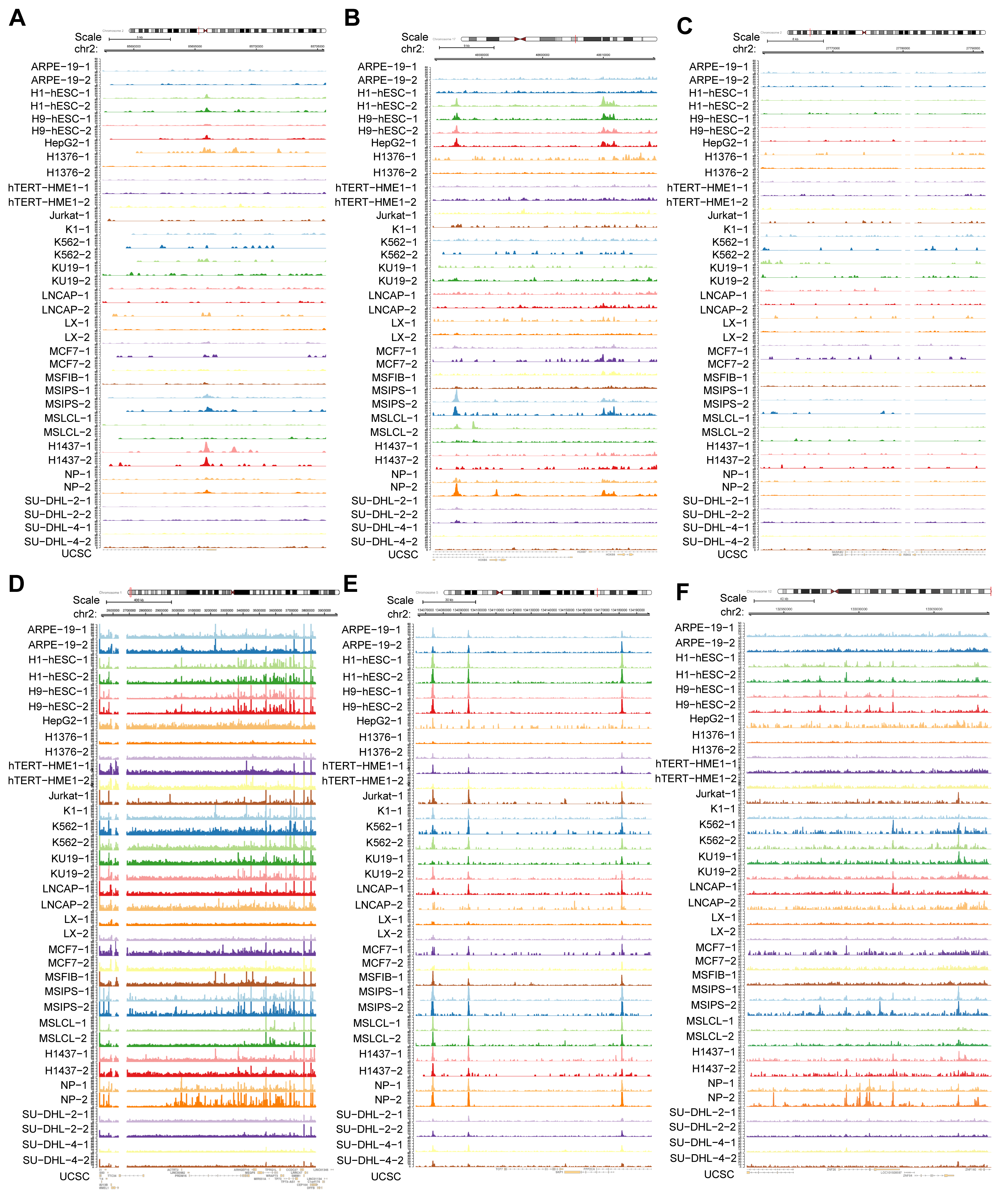

Supplement: Supplementary Figure 3 — Results of ATAC-seq analysis. The accessibility on chromatin of (A) GNLY, (B) HOXB7, (C) MRPL33, (D) TCF7 and (E) ZNF26 was verified by ATAC-seq analysis. [file Image_3.tif]

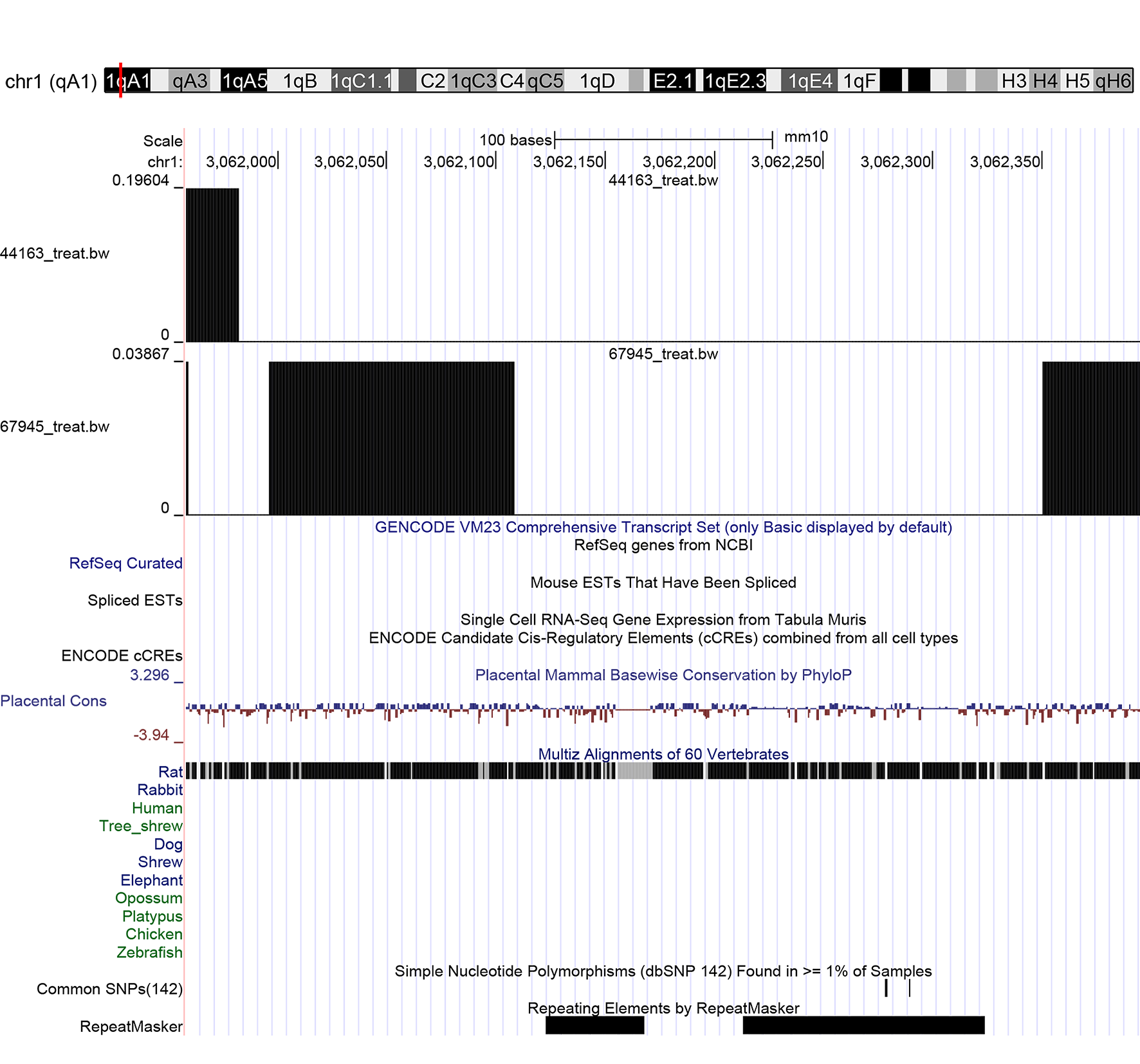

Supplement: Supplementary Figure 4 — The binding level between PRDM16 (eRNA) and ELK4 (TF) in response to the CHIP-seq analysis. [file Image_4.tif]

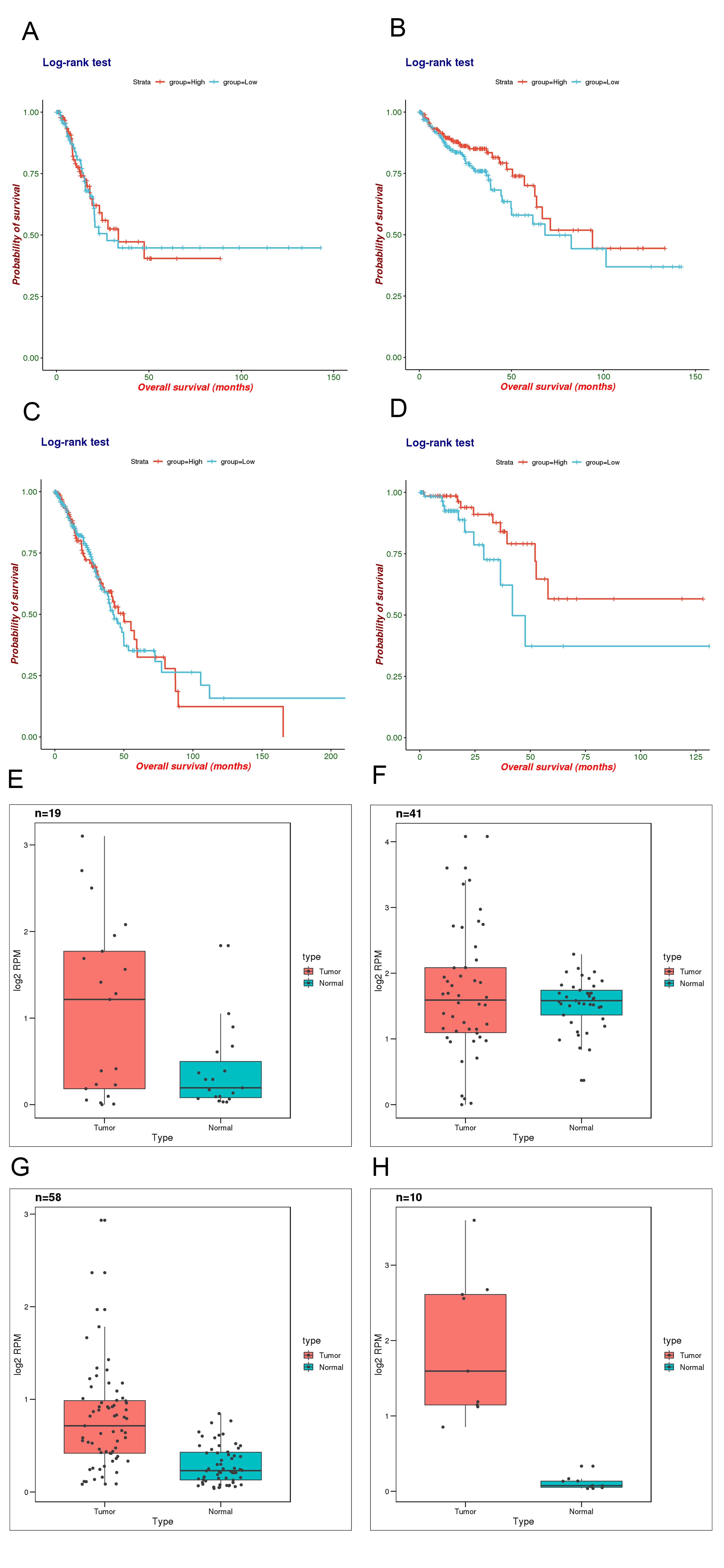

Supplement: Supplementary Figure 5 — The expression level of (A) GNLY, (B) HOXB7, (C) MRPL33 and (D) PRDM16 between the PitNETs and normal samples based on the eRic database. Kaplan Meier curves (KM_CURVE) of overall survival of (E) GNLY, (F) HOXB7, (G) MRPL33 and (H) PRDM16 based on the eRic database. [file Image_5.tif]
